# Wolf Population Size and Composition in One of Europe's Strongholds, the Romanian Carpathians

**DOI:** 10.1002/ece3.71200

**Published:** 2025-04-15

**Authors:** Ruben Iosif, Tomaž Skrbinšek, Nándor Erős, Marjeta Konec, Barbara Boljte, Maja Jan, Barbara Promberger‐Fürpass

**Affiliations:** ^1^ Foundation Conservation Carpathia Brașov Romania; ^2^ University of Ljubljana, Biotechnical Faculty Department Of Biology Ljubljana Slovenia; ^3^ DivjaLabs Ltd. Ljubljana Slovenia; ^4^ Centre for Ecological Research Institute of Aquatic Ecology Debrecen Hungary; ^5^ Centre for Systems Biology, Biodiversity and Bioresources, Hungarian Department of Biology and Ecology Babeş‐Bolyai University Cluj‐Napoca Romania

**Keywords:** *Canis lupus*, noninvasive DNA sampling, population abundance, population density, spatial capture–recapture

## Abstract

Strategies of coexistence with large carnivores should integrate scientific evidence, population monitoring providing an opportunity for advancing outdated management paradigms. We estimated wolf population density and social dynamics across a 1400 km^2^ area in a data‐poor region of the Romanian Carpathians. Across three consecutive years (2017–2018 until 2019–2020), we collected and genotyped 505 noninvasive DNA wolf samples (scat, hair and urine) to identify individuals, reconstruct pedigrees, and check for the presence of hybridization with domestic dogs. We identified 27 males, 20 females, and one F1 wolf–dog hybrid male. We delineated six wolf packs, with pack size varying between two and seven individuals, and documented yearly changes in pack composition. Using a spatial capture–recapture approach, we estimated population density at 2.35 wolves/100 km^2^ (95% BCI = 1.68–3.03) and population abundance at 70 individuals (95% BCI = 49–89). Noninvasive DNA data collection coupled with spatial capture–recapture has the potential to inform on wolf population size and dynamics at broader spatial scales, across different sampling areas representative of the diverse Carpathian landscapes, and across different levels of human impact, supporting wildlife decision making in one of Europe's main strongholds for large carnivores.

## Graphical abstract

Noninvasive DNA monitoring of wolf packs in the Romanian Carpathians revealed pack composition changes from 1 year to another. Using a spatially explicit capture–recapture framework, we estimated population density at 2.35 wolves/100 km^2^. Our results suggest that monitoring wolf population size and composition is feasible in the Romanian Carpathians via environmental DNA sampling, and we propose that wolf monitoring can be expanded to other areas representative of the diverse landscapes of the Romanian Carpathians.

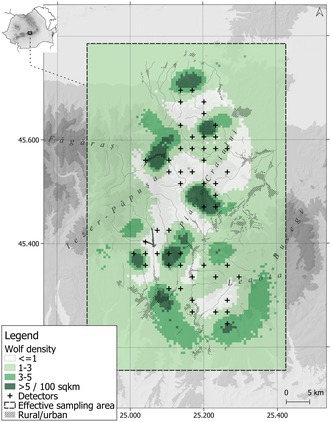

## 1 Introduction

Monitoring large carnivore populations is fundamental for responsible management strategies (Takinami et al. 2021), particularly when the paradigm changes from controlling populations to coexistence with humans (Creel and Rotella 2010; Bergstrom 2017). Population estimates guide scientists and practitioners to build coexistence actions, establish interventions, review their efficiency and impact on conservation goals (Fernández‐Gil et al. 2016; Lorand et al. 2022), and alleviate misconceptions around large carnivores in media and political discourse (Neagu et al. 2022). Reliable wildlife population estimates have the potential to inform and reshape management from a reactive approach characterized by poor data (Popescu et al. 2016) and weak institutional collaboration (Hartel et al. 2019) to a proactive approach where population data are transparently used to engage stakeholders in decision making (Can et al. 2014; Redpath et al. 2017).

Wolf ( *Canis lupus* ) is an apex predator with top‐down cascading effects on lower trophic levels (Vucetich et al. 2002; Hoeks et al. 2020). Usually, wolves live in packs with well‐defined territories (Mech and Boitani 2003). Wolf population sizes vary naturally, mostly with prey availability, and populations subjected to harvesting do not typically reach their full population potential (Fuller et al. 2003). The mating strategy of wolves influences pack composition, while the dispersal of individuals that join neighboring packs or create new ones maintains the genetic diversity and long‐term viability of wolf populations (Mech and Boitani 2003). In well‐connected landscapes, pack structure can be influenced by natural dispersal, but human‐caused mortality can severely disrupt kin‐based social structure (Rutledge et al. 2010), and may impact social group persistence, reproduction, and population dynamics (Fuller et al. 2003; Borg et al. 2015). The conservation status and the challenges of coexisting with wolves vary across the species range (Convention on the Conservation of European Wildlife and Natural Habitats 2022). Southern and Western European wolf populations are under threat from direct persecution, road mortality, or habitat loss at higher magnitudes than the northeastern populations (Chapron et al. 2014; Hindrikson et al. 2017). In the traditional agricultural landscapes of Eastern Europe, rapid socioeconomic change and shifts in human demography challenge coexistence with farmers due to loss of traditional conflict prevention knowledge (Kikvidze and Tevzadze 2015). Wolf–dog hybridization is another major issue for wolf conservation, and although it is recognized as being widespread across many areas of Europe, it is alarmingly unaddressed in practice (Salvatori et al. 2020; Stronen et al. 2022). To address these challenges, any coexistence strategies aimed at balancing the interests of humans without threatening the viability of wolf populations must be based on robust population and hybridization monitoring (Artelle et al. 2014). But assessing wolf population size, pack composition, and hybridization with dogs is a difficult task given the complex ecological mechanisms and life‐history traits of wolves, coupled with normally occurring low densities, elusive behavior, and large movement capability of the species.

Noninvasive DNA sampling, with DNA extracted from hair, scat, or urine deposited by animals in their habitats, has emerged as an effective tool in large carnivores monitoring and conservation (Schwartz et al. 2007). This approach has been refined to be faster and more cost‐effective than other (invasive) methods, and can be integrated into monitoring programs generating long‐term data (Kelly et al. 2012). In the case of a social animal like the wolf, in addition to density and sex ratio estimates, noninvasive DNA can provide additional inferences on family relations and hybridization with close taxa such as domestic dogs or coyotes (Caniglia et al. 2014; Kolenosky 1971; Stronen et al. 2022). Besides genetic variability and pack composition, successive individual genetic signatures (detections) of wolves can be developed into capture histories and modeled via capture–recapture methods to estimate population size and density (Pollock 2000; Williams et al. 2002). Spatial capture–recapture (SCR, Royle et al. 2014) overcomes the major shortcomings of regular or nonspatial capture–recapture: the uncertainty around estimating the effective sampling area (Efford 2004) and the spatially induced heterogeneity in encounter probabilities (Royle et al. 2018). SCR modeling has been used to estimate wolf population size and to assess spatial patterns of wolf density at different spatial scales across the western parts of the species range in North America (Roffler et al. 2019) and Western Europe (Jiménez et al. 2023; López‐Bao et al. 2018; Marucco et al. 2023; Mattioli et al. 2018).

The Romanian Carpathians sustain a viable population of wolves (Convention on the Conservation of European Wildlife and Natural Habitats 2022) with high genetic diversity (Ericson et al. 2020; Jan et al. 2023). However, climate change, habitat fragmentation, changes in agricultural practices, and shifts in the wildlife management approaches keep bringing new and more complex coexistence challenges (König et al. 2020). In the post‐socialist period, the Romanian Carpathians experienced changes in the traditional agricultural land use practices; small‐scale farming in remote areas was abandoned, potentially creating more habitats for ungulates closer to human settlements, while intensive agricultural practices and logging led to habitat fragmentation (Kuemmerle et al. 2009). Later, rapid human expansion and development of traffic networks, followed by uncontrolled tourism, further degraded forest habitats (Mustățea and Pătru‐Stupariu 2021). Cumulatively, we can expect these changes to have an impact on wolf dispersal, space use, natural prey availability, and conflict dynamics between livestock and wolves, but these impacts are not well understood. In 2016, the Romanian Government banned trophy hunting of wolves, which has been the traditional management strategy for decades. However, these management shifts did not include efforts to maintain and improve support for wolf conservation by various stakeholders, and monitoring has been less than adequate (Riener 2019). In this context, the Romanian Carpathians lack science‐based monitoring initiatives that would provide population size and pack composition data (Popescu et al. 2016), with the exception of only a few local initiatives that are trying to establish robust monitoring of wolf population size and structure in Romania (see Jarausch et al. 2023). The goals of this study were to estimate wolf population abundance and density in the Southern Romanian Carpathians and to assess changes in pack composition. We collected noninvasive DNA samples of scat, urine, and hair to identify individual wolves and reconstructed packs using parentage and sibship assignments across three consecutive years. We also assessed the magnitude of wolf–dog hybridization. Using SCR modeling, we estimated population abundance and density and assessed their spatial patterns. We aimed to provide not only a robust wolf population survey that would inform conservation and management actions but, more importantly, also a best‐practice example that could be used for scaling‐up of wolf monitoring in other parts of the Romanian Carpathians.

## 2 Materials and Methods

### 2.1 Sampling Design

**FIGURE 1:**
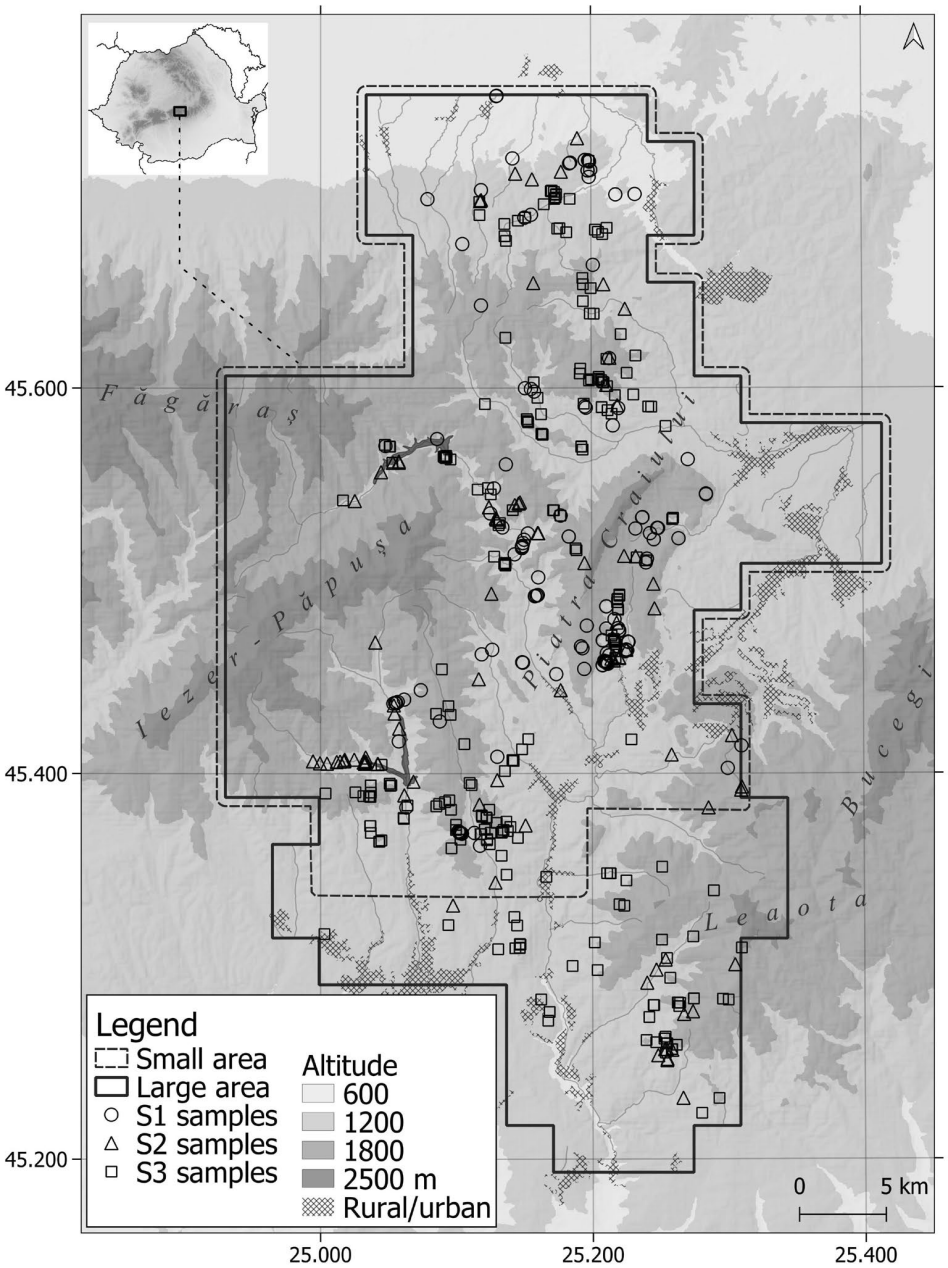
Sampling area for noninvasive DNA monitoring of wolf ( *Canis lupus* ) in Southern Carpathians, Romania. Map shows different symbols for the small and large sampling areas covered in different sampling years. In year 1 and 2 we sampled an area of 1100 km^2^ (Y1_*sa*_ and Y2_*sa*_) and the resulting data were used only for reconstructing the wolf packs. In year 3 the sampling area was expanded to 1400 km^2^ (Y3_*la*_) and we used the data for reconstructing the wolf packs and for estimating population abundance and density.

The sampling area is located in the Southern Romanian Carpathians, ranging in altitude between 600 and 2400 m (Figure 1). Deciduous, coniferous, and mixed forests cover 62% of the area in equal proportions. Mixed forests are located at mid‐elevations (around 1400–1500 m), with species composition dominated by beech‐fir or beech‐fir‐spruce *Fagus sylvatica* , *Abies alba* , and *Picea abies* communities. Beech dominates at lower elevations (900–1300 m), and conifers at higher elevations (1500–1800 m). Above 1800 m, the ecosystem mixes transitional woods with *Pinus mugo* , *Vaccinium subsp*. shrubs, and alpine grasslands. Lowlands are characterized by small‐scale farming and traditionally maintained mosaic landscapes (pastures, hayfields, and forests). Tourism is growing in the accessible areas, and livestock grazing that historically shaped the ecosystems in the alpine areas is still common nowadays. In the last three decades, the area was affected by forest clear‐cut operations, with logging still being an important economic activity. The sampling area harbors an intact mammal community that includes large and meso‐carnivores such as brown bear ( *Ursus arctos* ), wolf ( *Canis lupus* ), lynx ( *Lynx lynx* ), wildcat *(Felis silvestris)*, fox (*Vulpes vulpes*), and badger ( *Meles meles* ), as well as their prey: roe deer *(Capreolus capreolus)*, red deer ( *Cervus elaphus* ), chamois ( *Rupicapra rupicapra* ), wild boar ( *Sus scrofa* ) and leporids (e.g., hare, *Lepus europaeus* ). Characteristic for the area is also the high abundance of large carnivores, with lynx density estimated at 1.7 adults/100 km^2^ for example; Iosif et al. 2022). European bison ( *Bison bonasus* ) has also been recently reintroduced to the sampling area (Foundation Conservation Carpathia 2022).

The sampling was conducted over three consecutive years: 1 July 2017–31 June 2018 (year 1), 1 July 2018–31 June 2019 (year 2) and 1 July 2019–31 June 2020 (year 3). Between November and May, we actively searched for wolf tracks on snow 5 days per week to increase sampling success, while from May to November we sampled opportunistically when we identified fresh samples during other field activities. In year 1 and year 2, we sampled an area of 1100 km^2^. In year 3, this sampling area was expanded at the southern tip resulting in a larger area of 1400 km^2^. For simplicity, we will use subscript *sa* to refer to the smaller area (e.g., Y1_*sa*_), and the *la* subscript to refer to the larger area (e.g., Y3_*la*_), where Y and the number indicate the year in which the sampling was done (Figure 1). Samples across all years, independent on the moment of collection, were genotyped to identify individuals and reconstruct wolf families.

Prominent ridges, rivers (and adjacent logging roads), tourist routes, and visible animal paths were the primary target areas for sampling. If fresh wolf tracks were observed in the snow, the trail was backtracked until scat, hair, or urine samples were found. Other samples included blood on snow, saliva on carcasses of ungulates killed by wolves, and tissue samples from dead wolves (Table 1 and Appendix S2). Scat and urine samples estimated to be older than 5 days were not collected due to low expected genotyping success (Skrbinšek 2020). Sampling effort for each field day per ranger team was also recorded during year 3 as a GPS track, as these data have been used to carry out SCR analyses.

**TABLE 1:** Genotyping success by sample type across the three sampling years.

Sample type	Number of samples	Genotyped	Mixed	Poor	Nontarget
Scat	354	180	10	133	31
Urine	123	76	16	26	5
Hair	20	6	2	10	2
Blood‐noninvasive	4	3		1	
Saliva	2			2	
Tissue	2	1		1	
Total	505	52.7%	5.6%	34.3%	7.5%

*Note:* Genotyped samples refer to reliable wolf genotypes, mixed refers to DNA from two or more individuals detected in the same sample, poor refers to samples with insufficient quality, and non‐target refers to samples in which we found DNA belonging to other species such as fox and dog.

Collected hair samples from the field were shipped to a genetic laboratory (Dpt. of Biology, Biotechnical Faculty, University of Ljubljana, Slovenia) on a monthly basis to avoid DNA degradation, and DNA extraction was performed within a week of a sample's arrival to the laboratory. We stored scat and urine at −20°C until shipped to the genetic laboratory at the end of each sampling year.

### 2.2 Genotyping

We genotyped the samples at 16 canine unlinked autosomal microsatellite loci amplified in a single multiplex PCR and used the *Amelogenin* locus for sex determination (Appendix S2; Jan et al. 2023). DNA was extracted with the MagMAX Multisample Kit (ThermoFisher Scientific) using a liquid‐handling robot (Hamilton STARlet) to increase throughput and decrease the possibility of sample handling errors. To ensure reliable genotyping of noninvasive samples, we used a modified multiple‐tube approach (Taberlet et al. 1996; Skrbinšek et al. 2010). In the first screening, each sample was amplified with PCR and analyzed on an automatic sequencer (Applied Biosystem ABI 3130xl Genetic Analyzer) in two parallels. Samples that did not provide PCR products (amplification) were discarded; the samples that amplified were analyzed up to eight times. After each genotyping run, we checked genotype reliability using the Reliotype maximum‐likelihood approach (Miller et al. 2002) and calculated the quality index (Miquel et al. 2006) for each sample.

To identify samples matching the same genotype, we used a slightly modified procedure described by Skrbinšek (2020)). Since we used enough microsatellite markers relative to the expected number of animals in the sampling area, we allowed for some mismatches between samples. We allowed for one mismatch that could be caused by allelic dropout to still consider a match reliable, but did not allow mismatches that could be caused by false alleles (allelic incompatibilities between genotypes). To avoid problems with false alleles, we set a minimum threshold of two clear observations of an allele in separate analyses before the allele was considered “true.” We considered any matches with up to 3 possible allelic dropouts and 1 possible false allele mismatches as a “possible match” and collected further evidence (additional repeats) to either confirm or reject the match. If a genotype was reliably matched to another reliably genotyped sample, it was accepted even if the genotype reliability was below 0.98 threshold. If a sample was not matched to another reliable sample, the analysis was repeated until reliability reached the 0.98 threshold, or the sample was discarded after 8 replications if this threshold was not reached. When the quality index of a sample was below 0.4, the unmatched samples were discarded regardless of the estimated genotype reliability and considered the DNA quality too low.

### 2.3 Wolf–Dog Hybridization and Parentage Analysis

To assess wolf–dog hybridization, we used genotypes of 33 domestic dogs as a reference. We collected our reference dog samples during the same period and within the same sampling area as the wolf samples, to determine if the canids detected in the wild are pure wolves. We directly sampled domestic dogs using saliva swabs. We used Bayesian clustering in program STRUCTURE v. 2.3.4 to detect hybrids (Pritchard et al. 2000). Structure was run with 10^5^ iterations of burn‐in followed by 10^6^ Markov Chain Monte Carlo (MCMC) iterations. We used the population admixture model with correlated frequencies, used wild‐collected genotypes and reference dogs in the same run. We explored *K* = 2 and used CLUMPAK (Kopelman et al. 2015) to interpret results from independent runs. We used simulations to establish thresholds for detection of different levels of hybridization (see Appendix S3). As genetic structure in Carpathian wolves is not yet well understood and immigrants from other population clusters could easily be misidentified as hybrids (Ravagni et al. 2020), we did not consider hybrids further than the first generation wolf backcross.

Parentage and sibship assignments using genetic data enabled us to identify family groups and estimate the number of packs present in the sampling area. We used program Colony (Jones and Wang 2010) to simultaneously assign parentage and sibship assignments and determine family groups (packs) in the area. The Colony algorithm is particularly powerful since it enables both parentage and sibship assignments in the same model, providing more efficient use of available data. We allowed for a locus‐specific probability of an allelic dropout error on each locus (between 0.017 and 0.073) and a 0.004 probability of a false allele, so that the cumulative probability allowed for a genotype incompatibility on one locus. These locus‐specific probabilities were calculated as weights that added up to 1 using empirically detected locus‐specific single‐PCR error rates across parallel genotyping repeats. We performed 3 independent runs using full likelihood, medium precision, and long run.

Since the study included wolves from different litters and spanned several breeding seasons, assuming monogamy could introduce severe errors in the results. We made the final analysis with a model that relaxes the monogamy assumption and allows polygamy. Allowing polygamy in Colony models means that the models allow for half‐siblings (one parent different) which is realistic since wolves can have different mates during their lifetime when one of the pair dies. In most cases, a pack also includes the offspring of the mated pair, which might disperse over the course of the following years, but may be accompanied, at least temporarily, by wolves genetically unrelated to the pack (Pacheco et al. 2024). Genetically unrelated wolves can reproduce when one of the breeding pair is lost, but they can also reproduce with one of the offspring of a breeding pair, effectively creating two breeding pairs in the pack (Jędrzejewski et al. 2005). A quick check of the proximity of the samples of the unrelated individuals and the samples of the Colony‐related individuals in our dataset suggested that there were unrelated individuals traveling with established packs. Thus, we complemented the results of pedigree reconstructions by assigning unrelated individuals to a pack based on the proximity of the samples. We defined proximity as samples of unrelated individuals collected on the same day within 500 m (arbitrary threshold) from samples of any member of the pack, with the same field‐estimated age of the collected sample.

### 2.4 Population Estimates

Estimating population density via spatial capture recapture (SCR) requires recapture histories of individuals (one capture/mark and subsequent recapture events) and a spatial measure of sampling effort. While data from all sampling years were useful for understanding parental relations and changes in pack composition across time, for SCR population estimates we used only the Y3_*la*_ (large area) samples collected from November to May. This recapture dataset attempted to meet the population closure assumption required by SCR and had the sampling effort correctly recorded. Prior to summarizing the number of recaptures, we removed the spatially and temporally autocorrelated samples (samples of the same individual collected less than 1 km apart on the same day) to control for autocorrelation in individual detection probabilities (Dey et al. 2023). Due to long periods without snow cover, we had difficulties measuring potential sample detection covariates. For simplicity, we assumed equal detectability of scat, urine, and hair samples across all occasions of the SCR framework.

In this study, we used the Bayesian hierarchical MCMC model approach based on López‐Bao et al. (2018). The regular SCR model assumes that individual activity centers *i* = 1, 2,…, *N* are distributed over a region or state space *S* and that individuals were sampled by our detector array. The distribution of individual activity centers *s* = (*s*_*x*_, *s*_*y*_) was described in this study by a homogeneous point process, such that *s*_*i*_ ~ *Uniform*(*S*). The activity centers are latent variables to be estimated by the model for the *n* detected individuals at detectors *j = 1, 2,…, J* with locations *x*_*j*_. Assuming that detection frequencies are a decreasing function of the Euclidean distance *d*_*ij*_ between individual activity *s*_*i*_ and a detector location *x*_*j*_, the expected detection rate can be defined as:

$$\lambda_{\mathrm{ij}}=\lambda \left(s_{i},x_{j}\right)=\lambda_{0}\times \exp\left(\frac{-{d^{2}}_{\mathrm{ij}}}{2\sigma^{2}}\right)$$

where λ_0_ is the baseline encounter probability, *d*_*ij*_ is the distance between the *i* individual's activity center and *j* detector, while *σ* is the scale parameter of the half‐normal detection function, descriptive of the movement of the target species. We included the detector‐level covariates (in our case: sampling effort) in the baseline encounter probability:

$$\log\left(\lambda_{\text{0j}}\right)=\alpha_{0}+\alpha_{1}\times E_{j}$$

where *E*_*j*_ is the detector level sampling effort.

Finally, the number of times an individual occurs at a given detector is described by a Poisson distribution whose expected value is the *λ*_*ij*_ value shown above:

$$y_{\mathrm{ij}}\sim \text{Poisson}\left(\lambda_{\mathrm{ij}}\right).$$

We ran three MCMC chains with 1000 burn‐in steps and 50,000 iterations with a thinning rate of 5, resulting in 30,000 outputs. For checking the convergence of the MCMC chains, we visually inspected trace plots and used the Gelman–Rubin statistic *R‐hat* (Gelman and Rubin 1992), where values below 1.1 indicated good convergence (Appendix S1). The goodness of fit of models was tested threefold using Bayesian p‐values described in Royle et al. (2014): (i). individual encounter frequencies per detector, (ii). individual encounter frequencies aggregated for each individual; and (iii) detector frequencies aggregated for each detector (Appendix S1).

SCR models are used to estimate the density of wildlife populations using individual encounters at a spatial detector array within the effective sampling area, addressing uncertainty related to the edge effect. The state space *S* was chosen so that individuals with activity centres near the edge of the state space have a negligible probability of encountering each other, using ~3 × σ (Royle et al. 2014) (Appendix S1). We generated the detector array as follows: (1) we divided the sampling area into 2.5 × 2.5 km grid cells (Appendix S1) and counted the number of samples of each individual within each grid cell from November 2019 to May 2020. (2) The centroids of cells which contained genotyped samples were set as detectors within the SCR modeling. Finally, our SCR dataset consisted of individual encounters at each detector (Appendix S1). The sampling effort added in the model as a detector level covariate was measured as the actually traveled transect length within a 2.5 × 2.5 km grid cell (km × km^−2^). Our detector spacing after aggregation to the 2.5 × 2.5 km grid cell is less than 1.5 times the scale‐parameter of the detection function and should not impact on precision and accuracy of the SCR model (in the sense of Milleret et al. 2018 simulations on data aggregation with SCR). We opted for a finer resolution for aggregation compared to other SCR studies on wolves (i.e., 5 or 10 km) due to the highly fragmented mountain terrain that can restrict or direct movement of wolves, potentially biasing their detectability in space (Alexander et al. 2005; Milleret et al. 2018).

## 3 Results

### 3.1 Sample Collection and Genotyping Success

During the three sampling years, we collected 505 samples with the following distribution: 153 samples in the first year, 99 in the second year, and 251 in the third year. Of the total collected samples, 53% provided a reliable genotype of the target species (*genotyped samples*; Table 1); the further results are based on these samples. Samples with insufficient quality to provide a useful genotype (*poor samples*) represented 34%. DNA of two or more individuals collected in the same sample (*mixed samples*) represents 5.6% of the total samples, while 7.5% of the samples contained DNA from other species (*nontarget samples*; Table 1). Poor, mixed, and nontarget samples were excluded from further analyses. Urine samples had a higher amplification success than scat or hair samples (Table 1).

### 3.2 Population Composition

**FIGURE 2:**
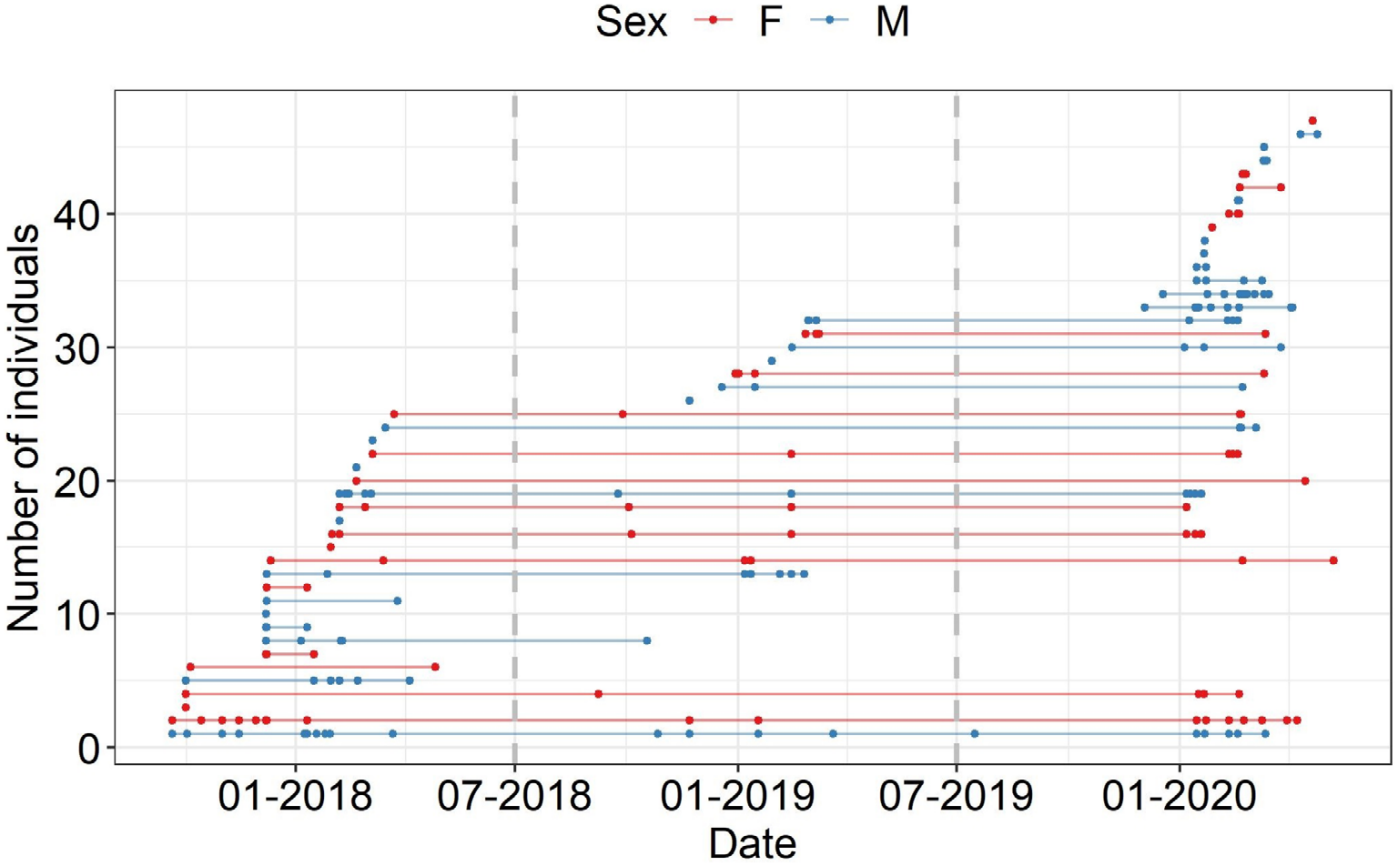
Recapture history of each individual wolf across the three sampling years in southern Carpathians, Romania.

Across the three sampling years, we identified 48 individuals: 27 males and 20 females plus one F1 wolf–dog hybrid male (qw = 0.51) (Figure 2). There were two other animals that indicated some possible dog ancestry at the level of the second backcross. We did not consider them hybrids since the (possible) hybridization level was low and could easily be an artifact of yet undescribed population structuring in the Carpathian wolves. In Y1_*sa*_, we detected 25 individuals. In Y2_*sa*_, we detected 18 individuals out of which 7 were new individuals, but missed 14 of the individuals detected in Y1_*sa*_. In Y3_*la*_, a total of 31 individuals were detected: 15 new individuals, 14 from Y1_*sa*_ and Y2_*sa*_, and 2 individuals missed in Y2_*sa*_ but captured again in Y3_*sa*_ (Figure 2). In Y3_*la*_, we detected 31 individuals, a higher number due to the larger sampling area, 17 being known from previous years (Figure 2). The mean recapture rate across years was 6.3 for males (SE = 1.2) and 4.75 (SE = 0.92) for females. The average recapture rate of reproductive individuals was 9 (SE = 2.28) while for offspring, it was 4.5 (SE = 0.64).

**FIGURE 3:**
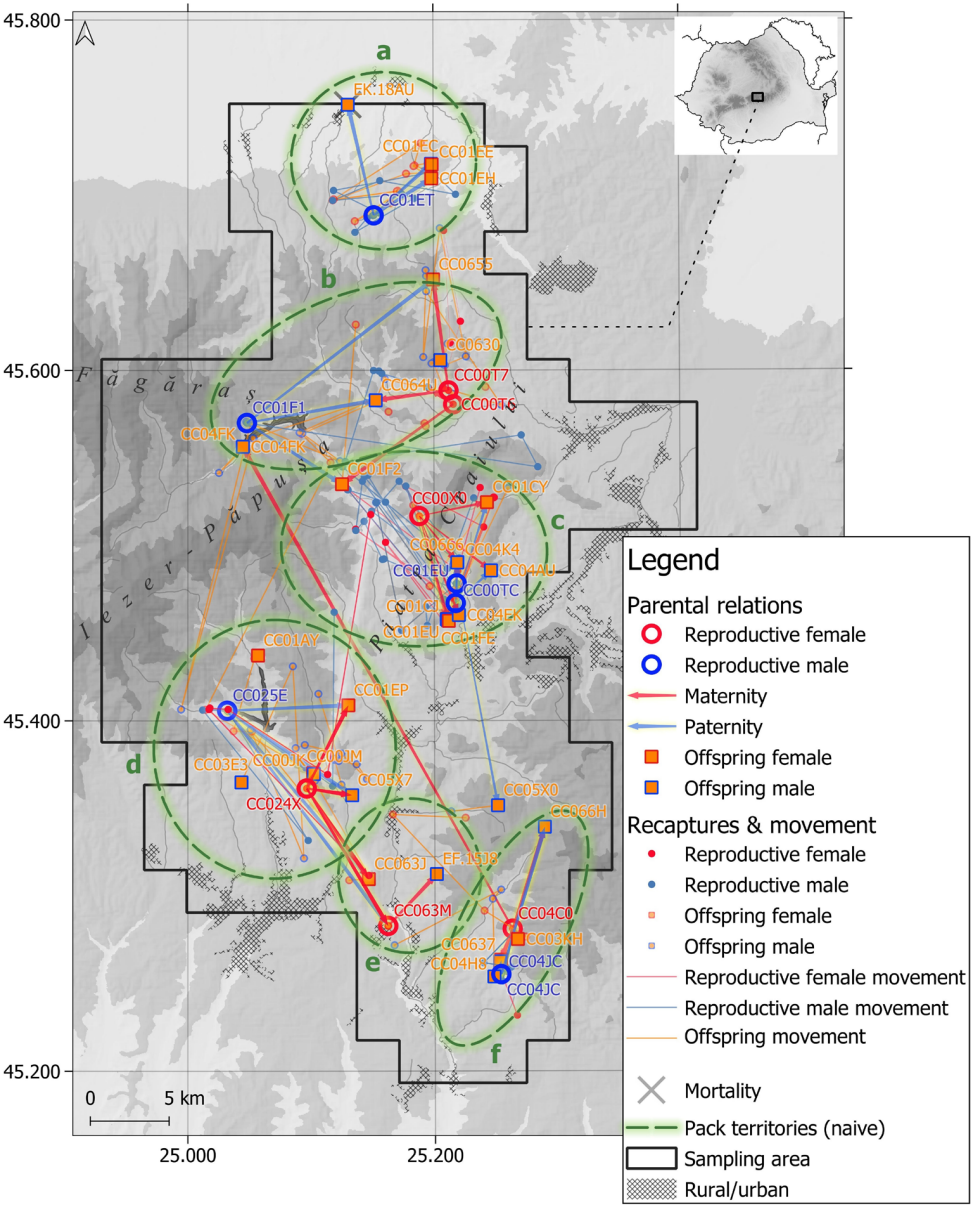
Parentage and sibship assignment with Colony method allowed us to reconstruct six wolf packs and track the shifts in the pack compositions in space. Pack territories were derived naively from parental relations and recapture and movement across the three sampling years. (a) refers to Sercaita pack, (b) to Dambovita–Barsa pack, (c) to Piatra Craiului pack, (d) to Dambovita–Raul Targului pack, (e) to Stoenesti hybrids, and (f) to Stoenesti pack.

**FIGURE 4:**
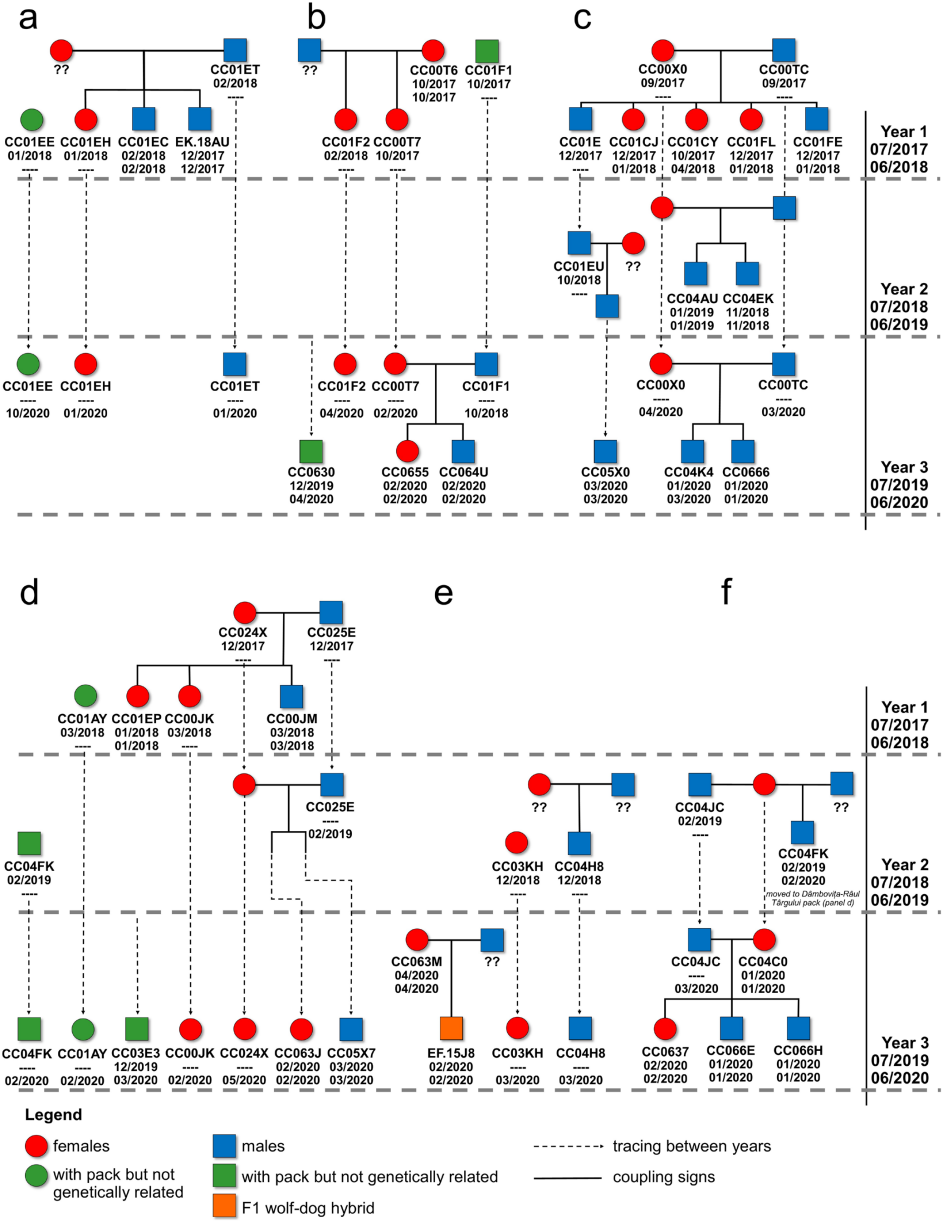
Parentage and sibship assignment with the Colony method allowed us to reconstruct six wolf packs and track the changes in the pack compositions over time. Under the symbol of each individual, we plotted the identification code and the collection date of the first and the last detection of that particular individual. Genetic relationships are marked across sampling years. (a) refers to the Sercaita pack, (b) to the DambovitaBarsa pack, (c) to the Piatra Craiului pack, (d) to the Dambovita–Raul Targului pack, (e) to the Stoenesti hybrids, and (f) to the Stoenesti pack.

While imperfect detection precluded us from identifying the full composition and structure of each wolf pack, we were able to assign 40 individual wolves and one wolf–dog hybrid into six packs (Figure 3 and Appendix S3). The remaining 7 wolves could not be assigned to any specific pack. Moreover, in three out of six packs, we identified individuals (*N* = 6) that were not genetically related to the pack but seemed to move with them as their samples were in close proximity to those of related individuals (range = 5–290 m). We assigned these individuals as members of the packs (Figure 4). Only one individual first detected in one pack later moved and traveled with another pack (Figures 3 and 4). Across all 3 years, we detected turnover in breeding pairs in 2 out of 6 packs (Figure 4 and Appendix S4). The first turnover was detected in the Dambovita–Barsa pack, where an unrelated male joined the pack in year 1 and replaced an undetected breeding male until year 3. The reproductive female remained the same across all years (Figure 4). The second turnover happened in the Stoenești hybrids pack when, in year 2, an undetected breeding pair produced two wolf offspring. In year 3, these two wolf offspring formed a pack with a newly detected breeding female wolf, an undetected reproductive male dog, and their F1 hybrid offspring. The Piatra Craiului pack maintained the reproductive pair across all years. In year 2, a male offspring from year 1 and an undetected female formed a second breeding pair that produced an additional male offspring. In year 3, the offspring of the second breeding pair merged with the individuals of the established pack while his father was no longer detected (Figure 4 and Appendix S4). The detected number of animals per pack ranged between 4 and 7 in year 1, 2 and 6 in year 2, and between 3 and 6 in year 3.

### 3.3 Demographic Estimates

**FIGURE 5:**
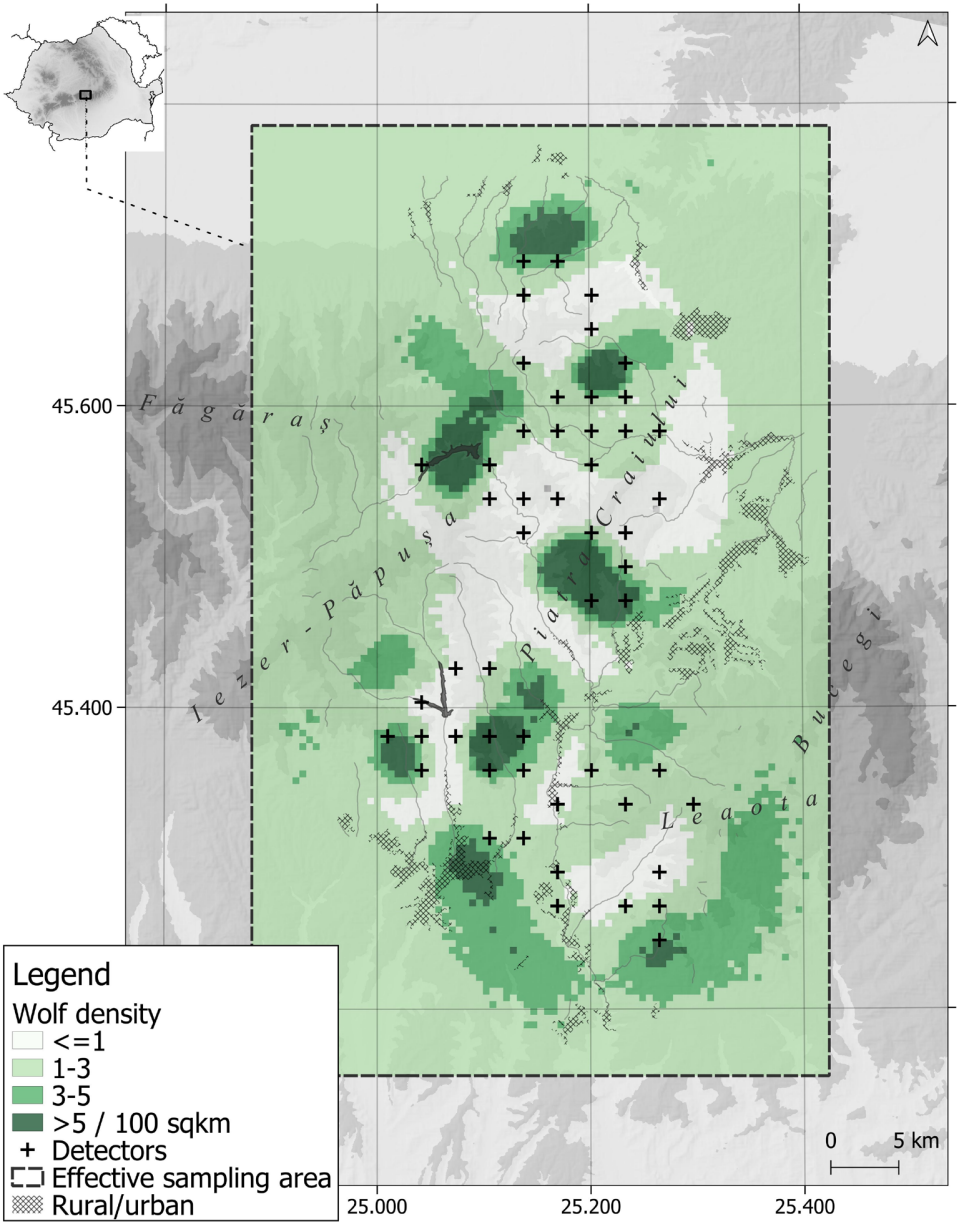
Posterior mean density of wolf activity centers reported for 100 km^2^. The detector locations used for interpolating the density surface are plotted on the map. The effective sampling area, 2975 km^2^, is obtained by buffering the σ spatial scaling parameter ~3 times around the detectors (see Methods).

Within the SCR framework, after applying a 10 km buffer (~3 × σ) around detectors, we used a state‐space area of 2975 km^2^. The posterior mean population size $\hat{N}$ in year 3 was 70 individuals (95% Bayesian Credible Interval; BCI = 49–89). The models revealed an even sex ratio with 33 females (BCI = 21–45) and 33 males (BCI = 21–45). The posterior mean density estimate ($\hat{D}$) in year 3 was 2.35 wolves/100 km^2^ (BCI = 1.65–2.99), comparable with other populations across the species range (Table 2). The posterior mean density of activity centers of wolves across the effective sampling area is presented in Figure 5.

**TABLE 2:** Pack size and density estimates in eight selected wolf populations across the species range and comparison with the current study.

Population	Study duration (years)	Method	Pack size	Density estimate (individuals or packs)	Reference
Polish Carpathians	5	Snow tracking	3.2–6.6	3.3–5.1 ind./100 km^2^	Śmietana and Wajda 1997
North‐west Poland	3	GPS/GSM telemetry, noninvasive DNA, camera trapping, howling and tracking	3.9–5.6	1.2 ind./100 km^2^	Mysłajek et al. 2018
Cantabrian	1	Noninvasive DNA	—	2.5 ind./100 km^2^	López‐Bao et al. 2018
Galicia	0.3	Camera trapping	6–16	2.5 ind./100 km^2^	Jiménez et al. 2023
Yellowstone National Park	7	Prey biomass proxy	—	3.4–9.8 ind./100 km^2^	Mech and Barber‐Meyer 2015
Apennine ‐ Central	2	Camera trapping and noninvasive DNA	3.4–4.2	1.2 packs/100 km^2^	Mattioli et al. 2018
Apennine ‐ North	8	Howling and tracking	4.2	4.7 ind./100 km^2^	Apollonio et al. 2004
Scandinavian	9	Noninvasive DNA	—	0.18 ind./100 km^2^	Milleret et al. 2021

*Note:* A mixture of methodologies was used to derive the parameters ranging from tracking to noninvasive DNA sampling. We selected populations from variable conditions, such as populations from biodiversity strongholds in North America, populations that are recovering from a recolonization bottleneck in Northern Europe, and populations in similar ecosystems in Eastern Europe.

## 4 Discussion

We provide an assessment of wolf population density in one of Europe's strongholds for this species, the Romanian Carpathians, using noninvasive DNA sampling and SCR modeling. We tracked 47 wolves and documented yearly changes in their pack composition. We show that noninvasive DNA monitoring can be successfully applied in the Romanian Carpathians to provide robust density estimates, including spatial variations in density, and to provide insights into some other parameters and threats critical for wolf conservation such as social dynamics and hybridization with domestic dogs.

Our results showed that the wolf density of 2.35 individuals/100 km^2^ (BCI = 1.65–2.99) we estimated for the Southern Romanian Carpathians is not higher than densities estimated in other parts of Europe (Table 2), with the exception of the Scandinavian population which lives in a different landscape and is recovering from a recolonization bottleneck, with current densities being less than 0.2 individuals/100km^2^ (Laikre et al. 2016; Milleret et al. 2021). One possible mechanism regulating wolf density in the Romanian Carpathians is related to interspecific interactions in an intact large carnivores' community (Dyck et al. 2022), as opposed to other mountain ranges in Western and Southern Europe—where other large carnivores have gone locally extinct. Bears may act as a regulating factor for wolf density. Ordiz et al. (2015) showed that Scandinavian wolf pair establishment was negatively correlated with bear population density, one possible explanation being that both species compete there, at least seasonally, for the same resources such as the ungulates. Unfortunately, and opposed to other places across the wolf range such as in North America (Hayes and Harestad 2000; Mech and Barber‐Meyer 2015; Serrouya et al. 2017), no robust data are currently available in Romania on competitors or prey numbers, posing difficulties in assessing the influence on our wolf population. Kleptoparasitism can be another possible mechanism regulating wolf density, with large amounts of competing apex predator kills being actually consumed by bears. For example, 50% of prey killed by Eurasian lynx were kleptoparasitized by bears in the Dinaric Mountains (Krofel et al. 2012). However, since wolves, especially established packs, consume prey much faster than lynx, we can assume this effect to be less important, further research being needed to understand bear–wolf interactions in our area. One uncertainty of our SCR application is not considering the gregarious nature of wolves, where the premises of uniform distribution and independence of detection between individuals may be violated. López‐Bao et al. (2018) suggested that spatial aggregation of their wolves resulted in only a slight underestimate of SCR density. Corroboratively, our goodness‐of‐fit tests show a rather small lack of fit, suggesting that pack behavior might not be highly gregarious at the time of our sampling or that cohesion might not be reflected by our sampling approach in the highly fragmented terrain (Appendix S1). However, Jiménez et al. (2023), using a regular SCR Poisson model on camera trap encounter histories of the Iberian wolves, experienced severe lack of fit. Moreover, Bischof et al. (2020) demonstrated that both the aggregation of activity centers (lack of spatial uniformity) and the cohesion or synchrony of detection patterns of members of a group (lack of detection independence) affected the accuracy of SCR model results in social animals. To deal with the aggregation and cohesion uncertainties, Jiménez et al. (2023) have proposed advances of the SCR application on wolves by comparing different observation models and using random effects in detection probability.

Our pack compositions changed from year to year with three detected dispersers between known packs, 7 individuals not assigned to a pack, and three packs accepting at least one individual not genetically related to the other members during our sampling. In general, this level of pack dynamics indicates good connectivity and gene flow between neighboring packs (Pacheco et al. 2024). Specific to the Romanian Carpathians, Ericson et al. (2020) confirmed a weak population differentiation in wolves, the traditional agricultural mosaic landscape favoring connectivity between wolf subpopulations. Similar inferences on habitat connectivity were made in the same area for Eurasian lynx, the well‐connected agricultural mosaic at lower elevation supporting lynx dispersal (Iosif et al. 2022).

We detected a turnover in breeding pairs in 2 out of 6 packs, with an apparent annual breeder mortality of 16% during the first and second reproductive seasons, suggesting a relatively high social stability in the short term. However, longer time frames should be considered for strengthening inferences on pack composition changes. Ausband et al. (2017) found that breeder turnover in wolves resulted in changes in group composition, further resulting in changes in the genetic content of a pack and impacting population growth. Borg et al. (2015) found that breeder loss preceded pack dissolution at the social unit level, especially when the breeder female or both breeders were lost. At the population level, however, wolves showed resilience mechanisms that compensate for disruption and harvesting by humans (Borg et al. 2015). Given that much of the wolf mortality across the species range is human‐induced (e.g., persecution through poisoning, competition between wolves and hunters and subsequent illegal shooting; Musto et al. 2021; Nowak et al. 2021, Riener 2019; Skogen 2022), and that mortality data are scarce in the Romanian Carpathians, long‐term monitoring of the Romanian wolf population should focus on density and social dynamics, but consider measures of apparent mortality as well.

Complementing the pedigree reconstruction with unrelated individuals helped us to improve inference on pack composition but also had limitations. We minimized errors by setting the distance between samples of unrelated vs. related individuals to less than 500 m and by accepting only samples with the same field‐estimated age. Our approach, however, could not account for errors associated with high variation in spatial movement of certain individuals (Gurarie et al. 2022), with marking in the same places by different packs or lone dispersers (i.e., marking intensity is known to increases in peripheral areas of pack territories; Zub et al. 2003), nor for the variability in movement patterns between breeders and nonbreeders (Demma and Mech 2009). We sometimes had difficulties in evaluating how many wolves traveled together due to unreliable conditions for snow tracking, thus making it difficult to evaluate whether unrelated wolves were actually accepted in the pack or just marking on top of other wolves. While our approach of complementing pedigree reconstruction with these data may induce a bias in the pack composition and pack size, it should impact less on the population density estimates.

We confirmed one F1 wolf–dog hybrid, to our knowledge the first genetic confirmation of a hybrid in the Romanian Carpathians. While there are two other individuals indicating a possible dog ancestry, we could not reliably classify them as wolf–dog hybrids with our marker system. Although hybridization in our population appears marginal at this time, an independent camera trapping study in the same study area and time frame showed that free‐ranging domestic dogs co‐occurring with wolves are a real concern (Iosif et al. 2022). Although temporal avoidance in the activity of dogs and wolves has been observed, our camera trap studies documented one free‐ranging dog encounter for every two wolf encounters in the wolf habitat in our area (Campbell 2021). In human‐dominated landscapes, habitat loss combined with a high presence of free‐ranging domestic dogs was shown to degrade the genetic composition of wolf populations, highlighting the need for maintaining large, well‐connected wolf populations in continuous landscapes to limit genetic introgression from domestic dogs (Pilot et al. 2021). In the fragmented landscape of central Italy, for example, hybrids formed 30.6% of the sampled wolf population, with the suspected actual rate of recent admixture being closer to 50% (Salvatori et al. 2019). In Galicia, NW Spain, hybrids represented 6% of the detected wolf population (Pacheco et al. 2017). More research is needed to understand wolf–dog hybridization in the Romanian Carpathians and to provide management solutions that would limit the presence and dispersal of free‐ranging dogs.

### 4.1 Coexistence and Management Implications

Our results suggest that monitoring wolf population and social dynamics, as well as hybridization with domestic dogs, is feasible in the Romanian Carpathians through use of multi‐year noninvasive DNA sampling. We showed that robust population estimates such as population size, number of packs, and local population density can be obtained in a single winter sampling session and with a sampling effort of ~180 samples over a 1000 km^2^ area unit, and that annual tracking of the pack dynamics could be done annually with a smaller sampling effort. The work power needed to collect ~180 samples over 1000 km^2^ is ~3 permanent, trained staff with an additional 3 volunteers during the winter sampling using two 4 × 4 cars to access the valleys. An estimate of the money needed to cover wolf monitoring 1 year over 1000 km^2^ is ~52,000 € and includes costs with genotyping the samples, salaries of the permanent staff, costs with volunteers, fuel and car maintenance as well as reporting and statistical analyses. The strength of our inferences could be increased when replicating or upscaling our study (i) through longitudinal sampling over longer periods of up to 10 years in targeted sampling areas considered at risk, especially with respect to pack dynamics due to human‐induced mortality, or wolf–dog hybridization, and (ii) through acknowledging in the modeling framework the gregarious nature of wolves. Future sampling should explore innovations such as expanding the identification of individuals from environmental DNA to increase sample size and amplification success, and reduce associated costs (e.g., DNA extracted from footprints in the snow; De Barba et al. 2024). This will allow annual repeats of the pedigree analysis and annual tracking of the pack dynamics with a low sampling effort and estimate population parameters with SCR by increasing the sampling efforts every 5 years. Our study has potential to support wolf management in the Carpathians by proving that the outdated method of counting tracks in snow currently deployed by the Romanian game wardens can be enhanced or replaced through use of noninvasive DNA. This is critical as snowpack has been decreasing (in depth and length of time) due to climate change, already making snow tracking impossible in some parts of the Romanian wolf range. Our findings show efficiency and challenges of a scientifically sound monitoring scheme for wolves in the Romanian Carpathians that could contribute to coexistence by facilitating engagement of local stakeholders, obtaining objective data, and helping policymakers to take efficient, science‐based conservation decisions.

## Author Contributions

**Ruben Iosif:** conceptualization (lead), data curation (equal), formal analysis (equal), project administration (equal), writing – original draft (lead). **Tomaž Skrbinšek:** conceptualization (lead), data curation (equal), funding acquisition (equal), methodology (equal), writing – review and editing (equal). **Nándor Erős:** formal analysis (equal), methodology (equal), writing – review and editing (equal). **Marjeta Konec:** formal analysis (equal), methodology (equal), writing – review and editing (equal). **Barbara Boljte:** formal analysis (equal), methodology (equal). **Maja Jan:** formal analysis (equal), methodology (equal), writing – review and editing (equal). **Barbara Promberger‐Fürpass:** conceptualization (lead), funding acquisition (lead), methodology (equal), project administration (equal), writing – review and editing (equal).

## Supporting information


**Appendix S1.** Spatial capture–recapture models for estimating wolf abundance and density in Southern Carpathians, Romania.

**Appendix S2.** Wolf DNA amplification success against year of sampling, month of sampling, and scat sample age in Southern Carpathians, Romania.

**Appendix S3.** Hybridization between wolves and dogs in Southern Carpathians, Romania.

**Appendix S4.** Performance of the parentage analysis and description of the pack compositions.

## Data Availability

The raw data used in this study as well as the scripts used for analyzing the data are available through the following link: https://zenodo.org/records/14544266?token=eyJhbGciOiJIUzUxMiJ9.eyJpZCI6IjhiMmM3YTMyLWViYzMtNDQzNy05MTRiLTVjOGViYzFmMzY5OSIsImRhdGEiOnt9LCJyYW5kb20iOiI3NjIyYjliMzVjMWM2YjA2MjM4ZDM0M2Y3YWM2NWZiMSJ9.4PQ0pE_AaHkNTe_fOe‐jC46fwGkgaAL2wtcUNMoWwHctdoDtK1rTzJm7pUtNi5OCHc7ShOFUYM1C2PaXf5C27Q
