# Supplementary material for: Wolf Population Size and Composition in One of Europe's Strongholds, the Romanian Carpathians
Source: Ecol Evol. 2025 Apr 15;15(4):e71200. doi: 10.1002/ece3.71200 (PMC12000540; doi:10.1002/ece3.71200)
Supplement: Supplementary file 1 — Appendix S1. Spatial capture–recapture models for estimating wolf abundance and density in Southern Carpathians, Romania. [file ECE3-15-e71200-s001.docx]

Appendix S1. Spatial capture recapture models for estimating wolf population abundance and density in Southern Carpathians, Romania.

*Model presentation*—The spatial capture recapture (SCR) models are used to estimate the population abundance or density of animals within an effective sampling area (Royle et al. 2013). The effective sampling area is a buffer around the detector grid to minimize the detection probability of animals outside the sampling area. SCR models typically require two input data: *i.* a dataset which describe the location of detectors and the detector level covariates (Figure 1), and *ii.* a dataset about detections of each individual at each detector (e.g., a N-by-M matrix where N is the number of individuals and M is the number of detectors; Figure 1).

Following López-Bao et al. (2018) we used a Bayesian Hierarchical Markov Chain Monte Carlo model approach that uses a spatial point process, i.e., randomly samples’ activity centers (coordinates) from the space and links the distance of locations to each detector via a detection function. We used half-normal detection function:

$$\lambda_{ij} = \lambda_{0}exp(\frac{{{-d}^{2}}_{ij}}{2\sigma^{2}})$$

where λ_0_ is the baseline encounter probability, d_ij_ is the distance between the *i* individual’s activity center and *j* detector while σ is the Gaussian scale parameter of the distance.

Detector level covariates, in our case the sampling effort (Figure 1) was linked to each observation:

log(λ_0j_) = α_0_ + α_1_×E_j_

where E_j_ is the detector level sampling effort.

Finally, the number of times an individual occurs at a given detector is described by a Poisson distribution whose expected value is the λ_ij_ value shown above:

y_ij_ ~ Poisson(λ_ij_).

We ran three MCMC chains with 1000 burn-in steps and 50,000 iterations with thinning rate 5, resulting in 30,000 outputs. For checking the convergence of MCMC parameters we used the Gelman-Rubin statistic *R-hat* (Gelman and Rubin 1992), where all values were below 1.1 thus indicating a good convergence. The goodness-of-fit of models were tested threefold using Bayesian p-values described in Royle et al. (2013): *i.* individual encounter frequencies per detector, *ii.* individual encounter frequencies aggregated for each individual; and *iii.* detector frequencies aggregated for each detector. The Bayesian p-value is calculated by the proportion of points above the 1:1 equality line. P-value between 0.05 and 0.95 indicates good fit, while 0.5 indicates perfect fit (Figure 2).


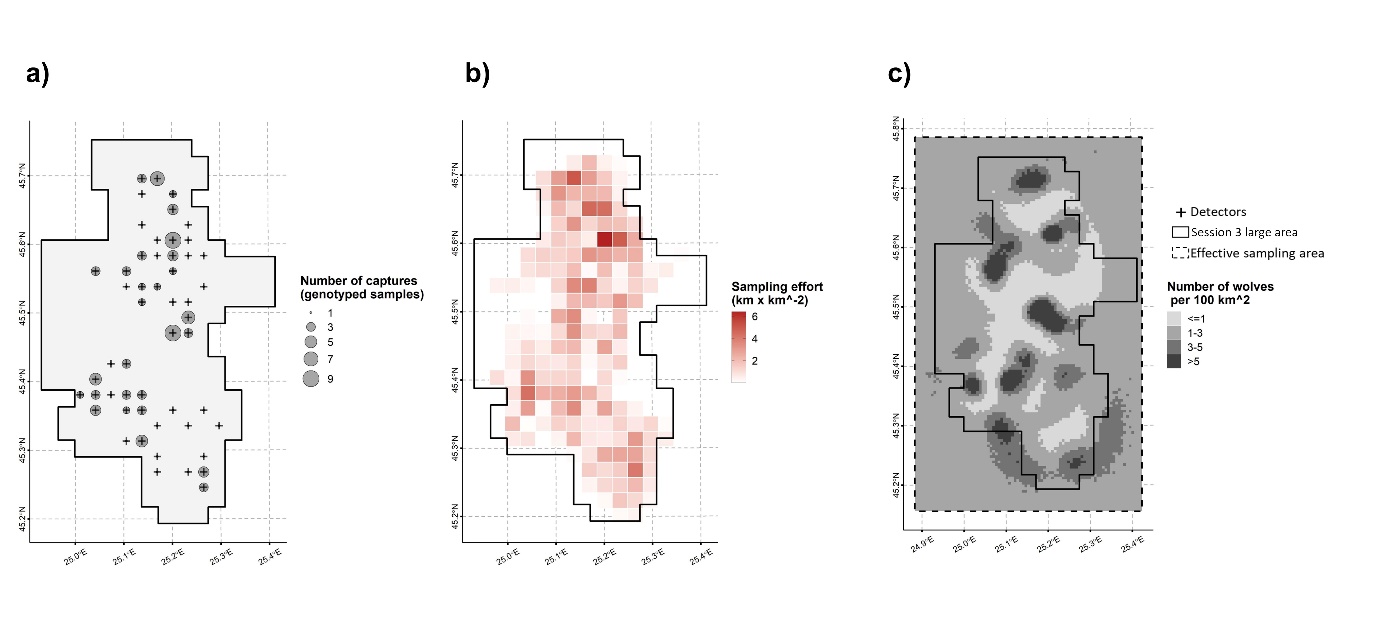


Figure 1. Panel a) Map of the sampling area with the number of captures, i.e., genotyped samples, collected at each detector (black crosses). Panel b) Sampling effort: the density of transect length (km × km^-2^). Panel c) The estimated wolf density within the effective sampling area.

*Model performance and result*—All the model parameter estimates were considered good based on R-hat statistics; all R-hat values were ≤ 1.1 (Table 1). The Bayesian p-values were between 0.05 and 0.95 indicating a good fit of the model (Figure 2). The Gaussian scale parameter (σ) had a posterior mean 0.332 (×10^4^ meters). This means that in the case when an individual’s activity center is 3320 meters from the detector gives a detection probability of 0.6 without taking into account the sampling effort dependent baseline encounter probability.

Table 1. Posterior mean estimates of the SCR model parameters to estimate wolf density. Alpha1 (α1) and alpha2 (α2) are the model parameters of the sampling effort covariate, psi (ψ) is the parameter of data augmentation and sigma (σ) is the Gaussian scale parameter of the half-normal detection probability function.

| **Scenario** | **Parameters** | **Mean** | **SD** | **Lower BCI (2.5%)** | **Upper BCI (97.5%)** |
| --- | --- | --- | --- | --- | --- |
| All | Density ($\hat{D}$) | 2.35 | 0.35 | 1.65 | 2.99 |
|  | Abund. (N) | 70 | 10.4 | 49 | 89 |
|  | alpha1 (α1) | -0.022 | 0.233 | -0.483 | 0.434 |
|  | alpha2 (α2) | 0.182 | 0.070 | 0.048 | 0.322 |
|  | psi (ψ) | 0.305 | 0.054 | 0.202 | 0.411 |
|  | sigma (σ) | 0.333 | 0.025 | 0.286 | 0.383 |
| Females | Density ($\hat{D}$) | 1.12 | 0.22 | 0.71 | 1.51 |
|  | Abund. (N) | 33.37 | 6.4 | 21 | 45 |
|  | alpha1 (α1) | 0.312 | 0.278 | -0.236 | 0.855 |
|  | alpha2 (α2) | 0.206 | 0.080 | 0.048 | 0.364 |
|  | psi (ψ) | 0.291 | 0.068 | 0.163 | 0.426 |
|  | sigma (σ) | 0.345 | 0.027 | 0.294 | 0.400 |
| Males | Density ($\hat{D}$) | 1.12 | 0.22 | 0.71 | 1.51 |
|  | Abund. (N) | 33.25 | 6.45 | 21 | 45 |
|  | alpha1 (α1) | 0.315 | 0.279 | -0.228 | 0.858 |
|  | alpha2 (α2) | 0.205 | 0.080 | 0.05 | 0.364 |
|  | psi (ψ) | 0.290 | 0.068 | 0.162 | 0.426 |
|  | sigma (σ) | 0.345 | 0.028 | 0.291 | 0.400 |


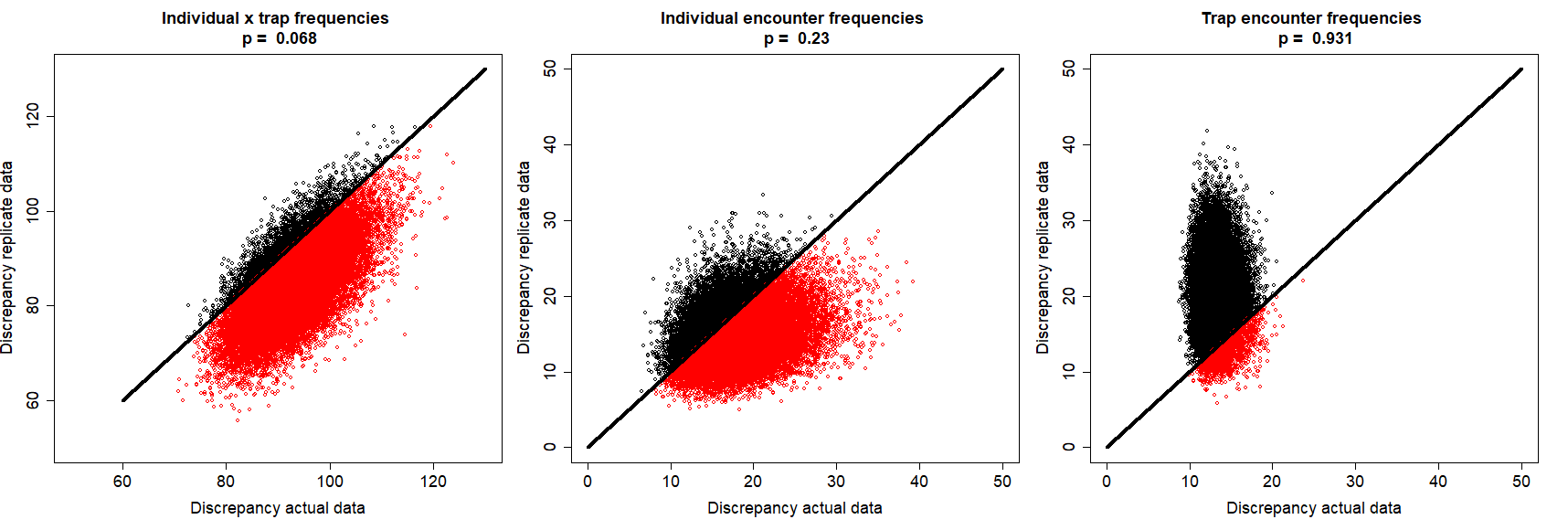


Figure 2. Discrepancy measures for SCR-Poisson Model used for SCR. Panel a) individual encounter frequencies per detector (p-value = 0.07); panel b) individual encounter frequencies aggregated for each individual (p-value = 0.24); panel c) detector frequencies aggregated for each detector (p-value = 0.93).

**LITERATURE CITED**

Gelman, A., Rubin, D. B. 1992. Inference from iterative simulation using multiple sequences. Statistical Science 7:457–472.

López-Bao, J. V., Godinho, R., Pacheco, C., Lema, F. J., García, E., Llaneza, L., Palacios, V. Jiménez, J. 2018. Toward reliable population estimates of wolves by combining spatial capture-recapture models and non-invasive DNA monitoring. Scientific Reports 8:1–8.

Royle, J. A., Chandler, R. B., Sollmann, R., Gardner, B. 2013. *Spatial capture-recapture*. Academic Press.
