# Supplementary material for: Wolf Population Size and Composition in One of Europe's Strongholds, the Romanian Carpathians
Source: Ecol Evol. 2025 Apr 15;15(4):e71200. doi: 10.1002/ece3.71200 (PMC12000540; doi:10.1002/ece3.71200)
Supplement: Supplementary file 2 — Appendix S2. Wolf DNA amplification success against year of sampling, month of sampling, and scat sample age in Southern Carpathians, Romania. [file ECE3-15-e71200-s002.docx]

Appendix S2. Wolf DNA amplification success against year of sampling, month of sampling and age of the sample (scat) in Southern Carpathians, Romania.

We backtracked wolves until scat, hair, or urine samples were found. Other samples included blood on snow, saliva on carcasses of ungulates killed by wolves, and tissue samples from dead wolves. Scat and urine samples estimated to be older than 5 days were not collected due to low expected amplification success. The hair sampling kits consisted of a paper envelope containing 10g desiccant (silica). The scat sampling kits consisted of 8 ml tubes, and the urine kit consisted of 50 ml tubes. Both types of tubes were pre-filled with 96% ethanol to preserve the collected samples. The scat and urine kits also contained two disposable wooden sticks that were used to collect the samples from the surface of the scat or from the snow. Another function of the sticks was to mark an already collected sample to avoid accidental re-sampling in the subsequent days. Each sampling kit (for collection of a single sample) was packed in a resealable bag fitted with a label on which we recorded the field data: the collector's name, date, GPS coordinates, field-estimated age of the scat, and a unique ID code for the sample. We collected additional data about location, habitat description, scat content, origin of a collected urine or hair (i.e., from the side of the road, from a fallen stump, etc.), as well as notes about the number of individual tracks seen in the snow, using a dedicated mobile app.

Across three years we collected 505 samples. Although the sampling was open year round, 90% of samples were systematically collected from November to May (Figure 1). We had the highest proportion of genotyped samples in January and February, while poor samples were frequent in March (Figure 1), suggesting that a systematic sampling during winter results in a higher genotyping rate. We genotyped the samples at 16 canine unlinked autosomal microsatellite loci: AHT137, AHTh171, AHTh260, AHTk211, AHTk253, CXX279, FH2054, FH2848, INRA21, INU030, INU055, REN162C04, REN169D01, REN169O18, REN247M23, REN54P11. Strict regimes were implemented for sample handling and analysis in the lab to avoid contamination, including a dedicated laboratory for pre-PCR handling of noninvasive genetic samples with strict contamination prevention protocols.


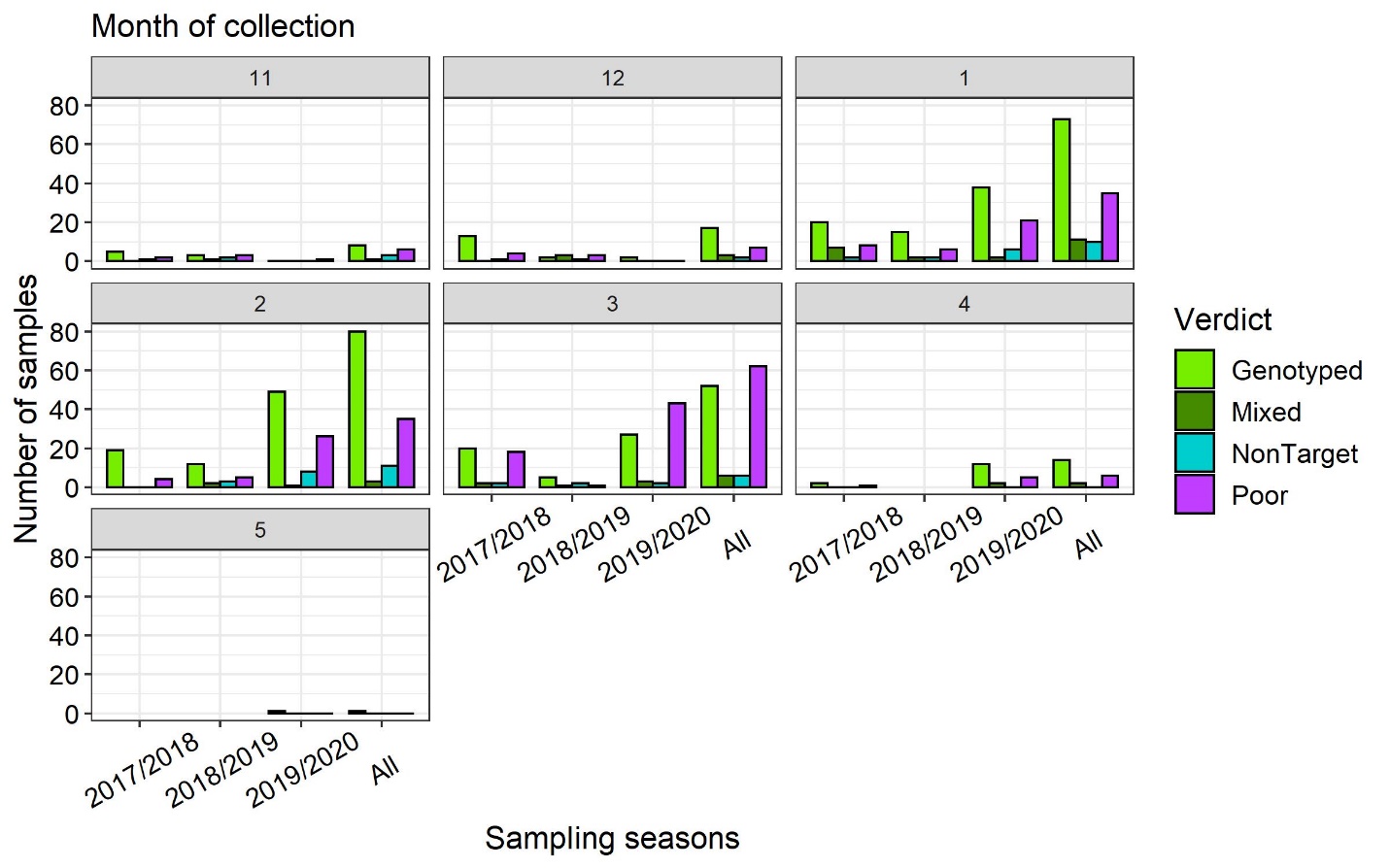


Figure 1. Amplification success of samples collected in different months across three years of sampling. Panels represent November (11), December (12), January (1), February (2), March (3), April (4) and May (5). On the X-axes the sampling years are marked.

Scat samples represented 70% of the total amount of samples, out of which 52.7% gave reliable genotype. The mean field-estimated age of scat samples was 2 days (SE = 0.23) in year 1, 1.8 days (SE = 0.21) in year 2, and 2.7 (SE = 0.15) in year 3 (Figure 2). Overall, samples estimated in the field to be one and two days’ old worked better than samples four-five days old (Figure 2).


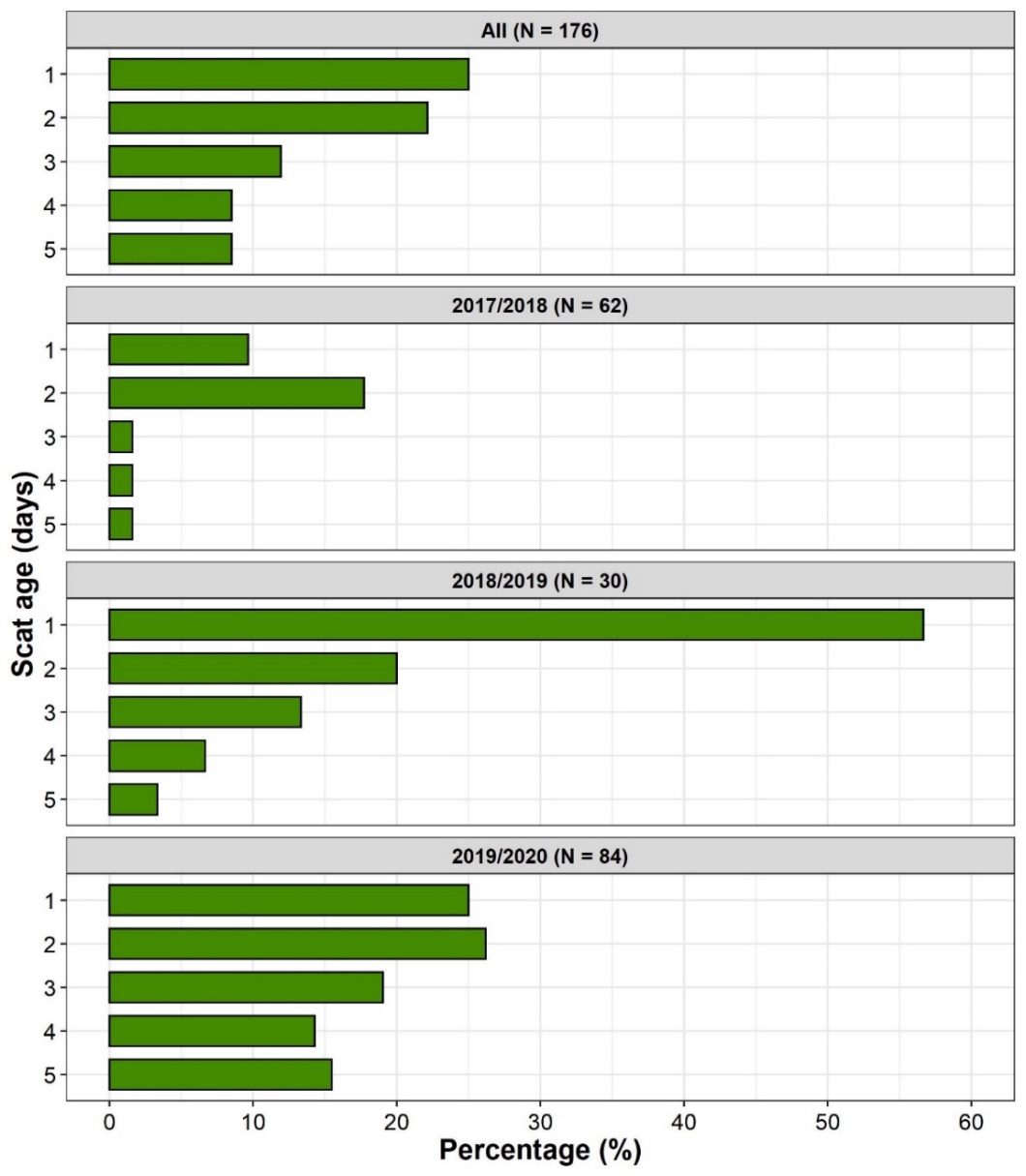


Figure 2. Scat samples amplification success by the field-estimated age of the samples (1-5 days old) in each year.
