# Supplementary material for: Wolf Population Size and Composition in One of Europe's Strongholds, the Romanian Carpathians
Source: Ecol Evol. 2025 Apr 15;15(4):e71200. doi: 10.1002/ece3.71200 (PMC12000540; doi:10.1002/ece3.71200)
Supplement: Supplementary file 3 — Appendix S3. Hybridization between wolves and dogs in Southern Carpathians, Romania. [file ECE3-15-e71200-s004.docx]

Appendix S3. Hybridization between wolves and dogs in Southern Carpathians, Romania.

We used Bayesian clustering in program STRUCTURE v. 2.3.4 to detect hybrids. In the same run we used the population admixture model with correlated frequencies and used wild-collected genotypes of wolves and 33 reference dogs sampled from the same sampling area. We used large-sized domestic dogs as a reference. Structure was run with 10^5^ iterations of burn-in followed by 10^6^ MCMC iteration. We used the population admixture model with correlated frequencies, used wild-collected genotypes and reference dogs in the same run. We explored K = 2 and used CLUMPAK to interpret results from independent runs (Figure 1).

To understand the threshold q-values for different levels of hybridization, we used R package Adegenet (Jombart 2008) to simulate F1 hybrids, and first and second generation backcrosses to wolves, 50 individual genotypes for each hybridization class. We used the genotypes of reference dogs to generate these hybrids. In wolf genotypes used for simulations, we excluded the animals that indicated possible dog ancestry in the STRUCTURE analysis (q value for belonging to the dog cluster >= 0.05). We also did a PCA analysis where wolves and dogs clearly arranged along the first component (also the only one informative according to the scree plot) and removed the presumed (field collected) wolf genotypes above the 80^th^ quantile (Figure 1). Simulated data, together with the wolf and dog data used to generate them, were analyzed in STRUCTURE with the same parameterization as described above. The results are shown in Figure 2, 3 and Table 1.

As thresholds to identify the hybrids, we used the 5^th^ quantile for the hybridization class (Table 1). Genotype EF.15J8, belonging to a male sampled in the wild, clusters clearly as a F1 wolf-dog hybrid (qw = 0.51). There are two additional animals that cluster as possible 2nd backcross hybrids (CC02H8, FCC00H; qw = 0.90, qw = 0.91, respectively), with the q-values lower than the minimum observed for “pure” wolves (Figure 4). However, as we don’t have the reference genotypes from all surrounding populations and since there can also be yet undescribed small-scale population structure in the Carpathian population (as is, for example, the case in the Dinaric Mountains, Šnjegota et al. 2021), these individuals could be immigrants or descendants of immigrants from another wolf population, and falsely detected as 2^nd^ generation backcross hybrids (Ravagni et al. 2020).


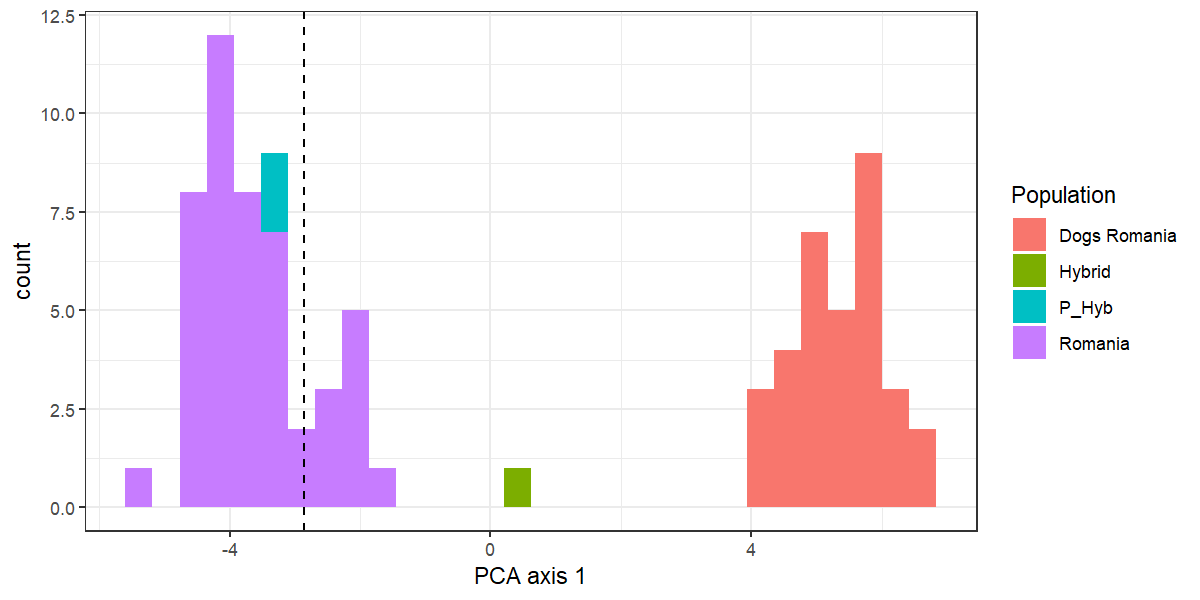


Figure 1: PCA analysis, arrangement of individual genotypes along the first PCA axis. Field collected samples above the 80th quantile (dashed vertical line) were not used for generation of the simulated hybrid genotypes, as were not the clear F1 hybrid and two individuals with possible dog ancestry (P_Hyb, q value for dog cluster >= 0.05).


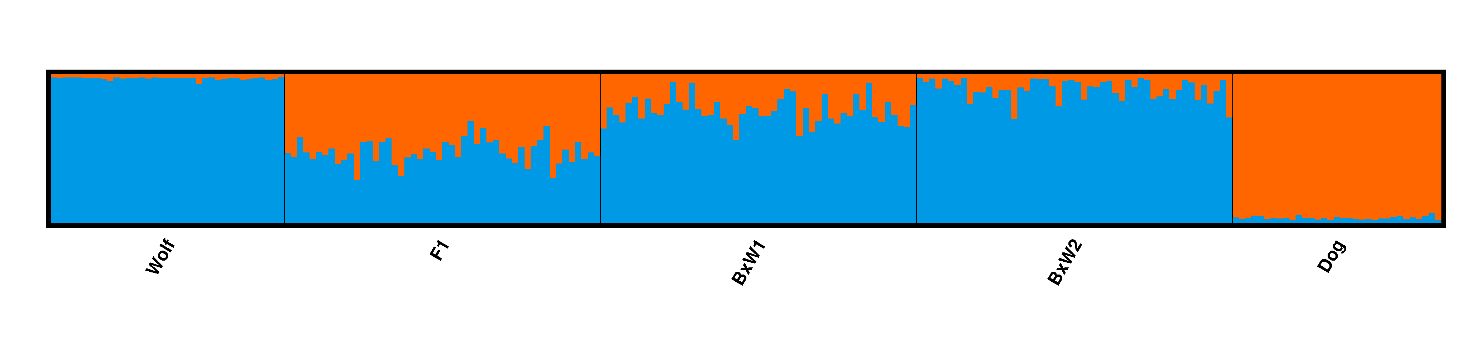


Figure 2: STRUCTURE plot of the analyzed simulated hybrid genotypes. Wolf and dog genotypes are the actual genotypes used to generate simulated data. BxW1 = first generation backcross to wolves, BxW2 = second generation backcross to wolves.

Table 1: Results of STRUCTURE analysis of simulated data for wolf – dog hybrids. The values are q-values of an animal belonging to the wolf cluster.

| **Population** | **min** | **q005** | **mean** | **median** | **q095** | **max** |
| --- | --- | --- | --- | --- | --- | --- |
| Dog | 0.02 | 0.02 | 0.04 | 0.03 | 0.05 | 0.07 |
| F1 | 0.29 | 0.34 | 0.48 | 0.47 | 0.62 | 0.69 |
| BxW1 | 0.56 | 0.62 | 0.76 | 0.74 | 0.92 | 0.95 |
| BxW2 | 0.70 | 0.79 | 0.90 | 0.92 | 0.97 | 0.98 |
| Wolf | 0.94 | 0.96 | 0.97 | 0.98 | 0.98 | 0.98 |


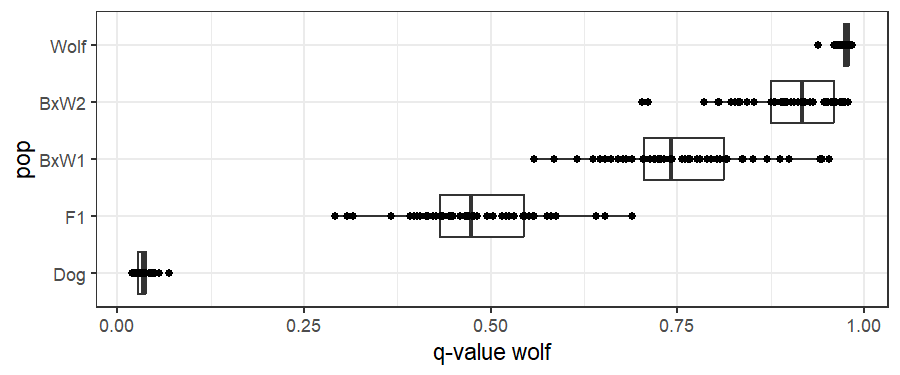


Figure 3: Results of STRUCTURE analysis of simulated data for wolf – dog hybrids. The values are q-values for an animal belonging to the wolf cluster. BxW1 = first generation backcross to wolves, BxW2 = second generation backcross to wolves.

**
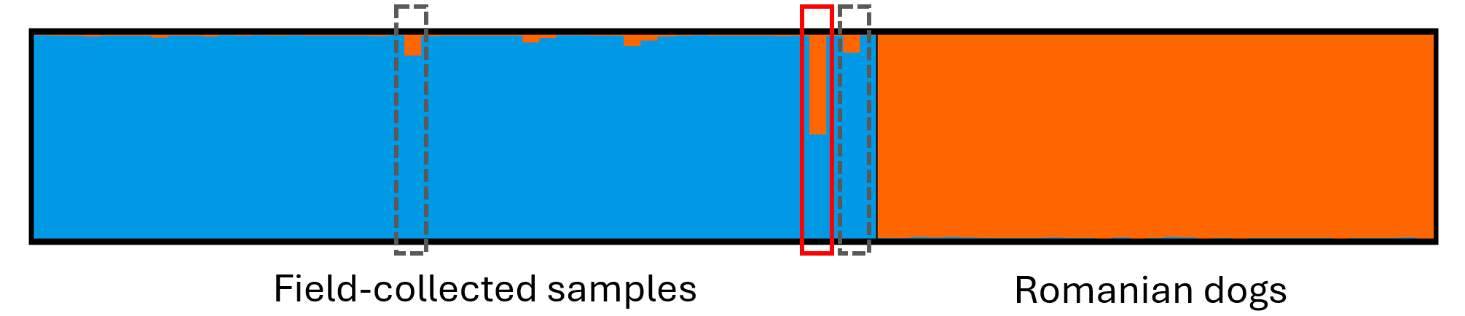
**

Figure 4. STRUCTURE graph of the wolf / dog hybridization analysis of the field collected samples and reference dog samples. Genotype EF.15J8, highlighted above with red full line and belonging to a male sampled in the wild, clusters clearly as a F1 wolf-dog hybrid (qw = 0.51). There are two additional animals (highlighted with dashed grey line) that cluster as possible 2*^nd^* backcross hybrids (CC02H8, FCC00H; qw = 0.90, qw = 0.91, respectively), however they may be immigrants or descendants of immigrants from another wolf population.

References:

Jombart, T. 2008. Adegenet: A R package for the multivariate analysis of genetic markers.

Bioinformatics, 24(11). https://doi.org/10.1093/bioinformatics/btn129

Šnjegota, D., Stronen, A. V., Boljte, B., Ćirović, D., Djan, M., Huber, D., Jelenčič, M., Konec, M., Kusak, J., & Skrbinšek, T. 2021. Population genetic structure of wolves in the northwestern Dinaric-Balkan region. Ecology and Evolution, 11(24), 18492–18504.

Ravagni, S., Sanchez-Donoso, I., & Vilà, C. 2020. Biased assessment of ongoing admixture using STRUCTURE in the absence of reference samples. Molecular Ecology Resources, 21, 677 - 689.
