# Supplementary material for: Wolf Population Size and Composition in One of Europe's Strongholds, the Romanian Carpathians
Source: Ecol Evol. 2025 Apr 15;15(4):e71200. doi: 10.1002/ece3.71200 (PMC12000540; doi:10.1002/ece3.71200)
Supplement: Supplementary file 4 — Appendix S4. Performance of the parentage analysis and description of the pack compositions. [file ECE3-15-e71200-s003.docx]

Appendix S4. Performance of the parentage analysis and description of the pack compositions.

Parentage analysis was done in program Colony. We allowed for a locus-specific probability of allelic dropout error on each locus (between 0.017 and 0.073) and 0.004 probability of a false allele. We performed 3 independent runs using full-likelihood, medium precision and long run. While the first analyses were run assuming monogamy, the results didn't provide correct pedigree assignments. When analysis was run using a polygamy assumption, the results and their interpretation improved. We checked the performance of the Colony run using traceplots of MCMC chains, with converged at the same likelihood level indicating decent convergence (Figure 1).

**
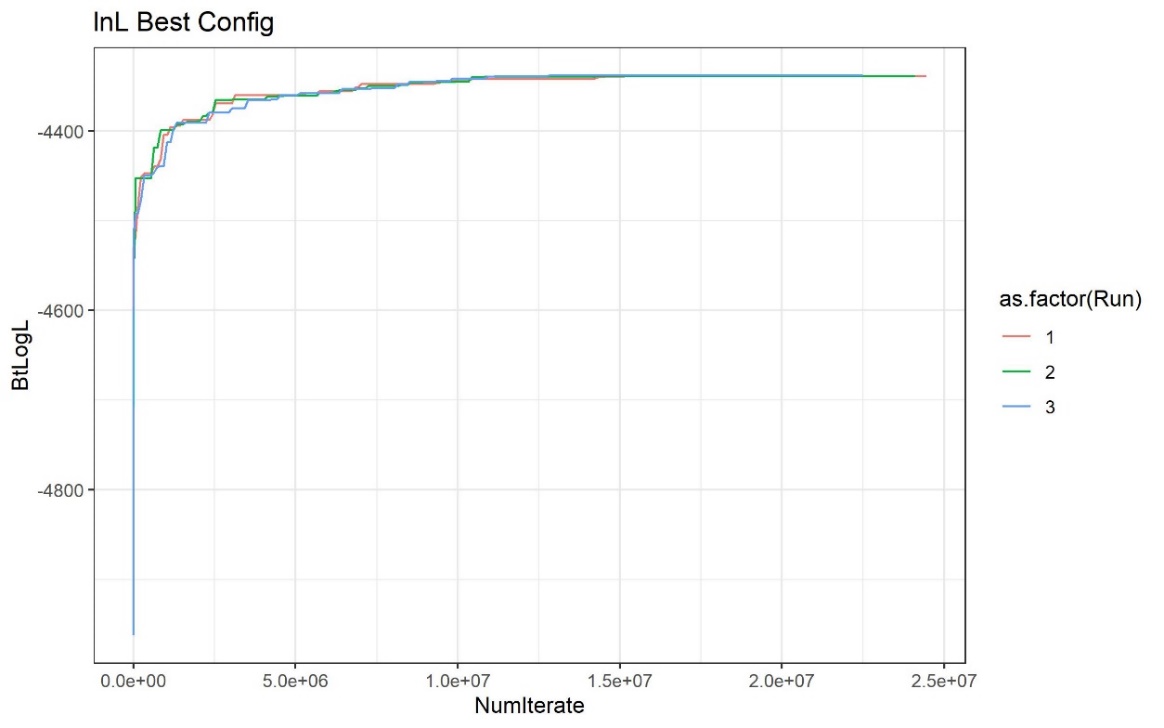
**

Figure 1. Traceplot of three MCMC Chains used for parentage analysis in Colony software.

We checked the stability of the parentage assignments also with traceplot, where all MCMC chains didn’t converge completely because of the presence of several single-parent-offspring pairs, which can go either way in different estimates. This later was probably the case since two of the three runs converge perfectly and one not (Figure 2).

**
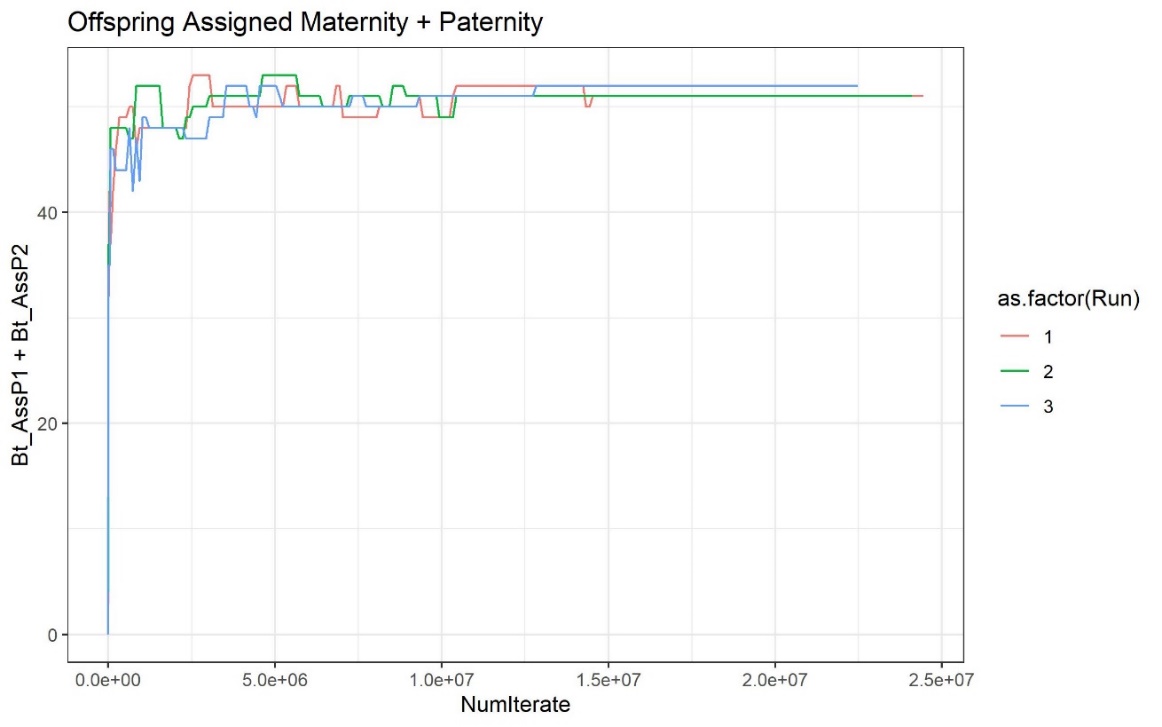
**

Figure 2. Stability of parentage assignments using three MCMC Chains.

*Sercaita pack*—Initially, this pack had four identified individuals with genetic relationship between them and a female that was not genetically related. During the year 2 and year 3 there was recaptures of the reproductive male (CC01ET), an offspring female (CC01EH) and the unrelated female (CC01EE).

*Dambovita – Barsa pack*—during year 1 this pack contained four genetically related individuals. The CC00T6 reproductive female had two offspring (CC01F2 and CC00T7) from an undetected male. CC01F1 male travelled with the pack although not genetically related. In year 3, CC01F1 male became a reproductive and had two offspring with an offspring female CC00T7 from year 1. CC01F2 offspring female from year 1 remained with the pack in year 3 as well. While CC01F1 male and CC00T7 female might have been reproductive pair in year 2 as well, we failed to sample them during year 2. CC01F1 made circular reference from year 1 to year 3. This happens in multi-generation pedigrees with limited sampling and some genotyping errors. A male (CC0630) traveled with the pack in year 3 although not genetically related with the other members of the pack.

*Piatra Craiului pack*—during the first year this pack contained seven individuals: two reproductive individuals and five offspring. During year 2 the pack attempted to split in two when CC01EU male became reproductive with an unknown female. In year 3 there were three offspring: two from the original reproductive pair and one from CC01EU and an undetected female. The offspring of the second reproductive pair was already detected exploring new territories in the south, outside of the initial pack territory.

*Dambovita – Raul Targului pack*—In year 1, this pack contained a reproductive pair, their three offspring and an unrelated female CC01AY. In year 2, this reproductive pair had two offspring: CC063J and CC05X7, kept offspring CC00JK from year 1 but skipped CC01EP and CC00JM. The pack in year 3 contained the reproductive female from year 1 and 2 (CC024X), the two offspring from year 2 (CC063J and CC05X7), a female offspring from year 1, CC00JK, and two genetically unrelated individuals CC03E3, CC01AY.

*Stoenesti hybrids*—Stoenesti hybrids pack was first detected in year 2, when included two pure yet unrelated wolves: male CC04H8 and female CC03KH. During year 3, together with the male and female wolves detected in year 2, we identified an F1 hybrid which was genetically related to a second wolf female CC063M and an undetected dog male.

*Stoenesti*—Stoenesti pack was first detected in year 2: a reproductive male and a polygamous female (captured in year 3). The reproductive female had an offspring male from an undetected male. Across years, this offspring male CC04FK left the pack and dispersed north to territories of *Dambovita – Raul Targului* and *Barsa – Izvoarele Dambovitei* packs, where he seems to remain a solitary individual. In year 3 the reproductive female had three offspring (a female and two males) with the reproductive male identified in year 1 (CC04JC).
